# MRI-Based Bone Marrow Radiomics Nomogram for Prediction of Overall Survival in Patients With Multiple Myeloma

**DOI:** 10.3389/fonc.2021.709813

**Published:** 2021-12-01

**Authors:** Yang Li, Yang Liu, Ping Yin, Chuanxi Hao, Chao Sun, Lei Chen, Sicong Wang, Nan Hong

**Affiliations:** ^1^ Department of Radiology, Peking University People’s Hospital, Beijing, China; ^2^ Peking University Institute of Hematology, Peking University People’s Hospital, Beijing, China; ^3^ Beijing Key Laboratory of Hematopoietic Stem Cell Transplantation, Peking University, Beijing, China; ^4^ Collaborative Innovation Center of Hematology, Peking University, Beijing, China; ^5^ Pharmaceutical Diagnostics, GE Healthcare, Shanghai, China

**Keywords:** multiple myeloma, magnetic resonance imaging, radiomics, nomogram, survival

## Abstract

**Purpose:**

To develop and validate a radiomics nomogram for predicting overall survival (OS) in multiple myeloma (MM) patients.

**Material and Methods:**

A total of 121 MM patients was enrolled and divided into training (n=84) and validation (n=37) sets. The radiomics signature was established by the selected radiomics features from lumbar MRI. The radiomics signature and clinical risk factors were integrated in multivariate Cox regression model for constructing radiomics nomogram to predict MM OS. The predictive ability and accuracy of the nomogram were evaluated by the index of concordance (C-index) and calibration curves, and compared with other four models including the clinical model, radiomics signature model, the Durie-Salmon staging system (D-S) and the International Staging System (ISS). The potential association between the radiomics signature and progression-free survival (PFS) was also explored.

**Results:**

The radiomics signature, 1q21 gain, del (17p), and β2-MG≥5.5 mg/L showed significant association with MM OS. The predictive ability of radiomics nomogram was better than the clinical model, radiomics signature model, the D-S and the ISS (C-index: 0.793 *vs*. 0.733 *vs*. 0.742 *vs*. 0.554 *vs*. 0.671 in training set, and 0.812 *vs*. 0.799 vs.0.717 *vs*. 0.512 *vs*. 0.761 in validation set). The radiomics signature lacked the predictive ability for PFS (log-rank *P*=0.001 in training set and log-rank *P*=0.103 in validation set), whereas the 1-, 2- and 3-year PFS rates all showed significant difference between the high and low risk groups (*P ≤* 0.05).

**Conclusion:**

The MRI-based bone marrow radiomics may be an additional useful tool for MM OS prediction.

## Introduction

Multiple myeloma (MM) is the second most common hematologic malignancy, characterized by anemia, hypercalcemia, renal failure, and lytic bone lesions (1). Despite the more effective therapies were introduced, this incurable disease remains highly heterogeneous in clinical outcome due to the patient characteristics and features intrinsic to the MM (2, 3). The challenge was that patients should accept the personalized intervention for both adequate quality life and prolonged survival. Therefore, accurate predicative markers for prognosis are needed to develop appropriate treatment in newly diagnosed MM.

Many factors including patient characteristics, disease biology and genetic lesions had the prognostic value that should be considered for patient assessment (4). Currently, several risk stratification models were routinely used in clinical practice, such as Durie-Salmon staging system (D-S) (5), the International Staging System (ISS) (6), the Revised-International Staging System (R-ISS) (7). and the Mayo Stratification of Myeloma and Risk-Adapted Therapy (mSMART) (8). However, these models should be further analyzed and refined, and the accurate prognostic stratification is still under research.

Imaging plays an important role in MM diagnosis and follow-up, the X-ray, CT, PET-CT and MRI were widely used in clinical practice. The X-ray is the most commonly used but difficult to detect lytic lesions (9). PET-CT has the ability to identify bone destruction and lytic lesions with assessment of tumor burden and disease activity (10). CT provides important information detecting bone destruction in particular the lesions in long bones (11). Compared with PET-CT and CT, the MRI has been considered as the most sensitive imaging method for detecting bone marrow infiltration, the normal, focal, diffuse, combined focal and diffuse, and variegated were five recognized patterns in MM (12, 13). Many studies reported the correlation between MRI and MM prognosis, suggesting the underlying ability of MRI for more accurate risk stratification (14–16).

Radiomics is an emerging field of research based on data-driven analysis of radiologic images, and it enables efficient elucidation of subtle characteristics within images that may provide clinically relevant information (17). Apparently, radiomics could be a potential tool for increasing the accuracy of the disease diagnosis, prognosis and treatment response assessment and further promoting the development of precision medicine. Recent years, many studies explored the capacity of radiomics in survival prediction in different types of cancers such as breast cancer, pancreatic cancer, lung cancer and nasopharyngeal cancer, and demonstrated the great value of radiomic analysis (18–21).

We speculate that the bone marrow MR radiomics may provide incremental information for survival prediction in patients with MM. Therefore, we constructed and validated radiomics nomogram for MM overall survival (OS) prediction, and compared it with other models. Additionally, the potential correlation between OS-based radiomics signature and Progression-free survival (PFS) was also explored.

## Materials and Methods

### Patients

This retrospective study was approved by the Institutional Ethics Committee in our hospital, and the informed consent requirement was waived. A total of 121 consecutive MM patients who underwent lumbar MRI at the initial diagnosis between January 2009 and November 2017 were enrolled. Inclusion criteria:1. Patients were diagnosed with MM according to the IMWG diagnostic criteria (1). 2. Complete baseline MRI included sagittal T1-weighted images (T1WI), sagittal T2-weighted images with fat suppression (T2WI-FS). 3. Complete clinical data of all patients were available. Exclusion criteria: 1. Patients diagnosed with monoclonal gammopathy of undetermined significance, smoldering MM and primary amyloidosis. 2. Patients had a previous history of receiving chemotheraphy or radiation therapy. 3. The images with obvious artifacts. 4. Patients that combined with other malignant diseases. The patient selection process was shown in **Figure S1**.

The follow-up information was acquired by the outpatient and inpatient medical records and telephone calls. Patients were followed until November 2020. OS was defined as the time from the date of diagnosis to death from any cause. PFS was defined as the time from the date of diagnosis to disease progression or death from any cause.

### MRI Protocol

Baseline imaging was all performed on a 1.5T MR image scanner (Signa Excite, GE Medical Systems). The scan parameters were listed as follows: sagittal T1WI: repetition time (TR) = 560 msec, echo time (TE) = 8 msec, slice thickness = 5 mm, matrix = 300 × 256, and field of view (FOV) = 32 × 32 cm; sagittal T2WI-FS: TR = 2500 msec, TE = 110 msec, slice thickness = 5 mm, matrix = 300 × 256, and FOV = 32 × 32 cm.

### Image Preprocessing and Segmentation

The T1WI and T2WI-FS Digital Imaging and Communications in Medicine images were exported from the Picture Archiving and Communication System. Then the data were preprocessed by using Artificial Intelligence Kit software version 3.3.0 (AK, GE Healthcare, China), including resampling the image into 1 × 1 × 1 mm^3^, signal smoothing by a Gaussian filter with the standard deviation of 0.5, bias field correction and intensity standardization by z-score transformation.

**Figure 1:**
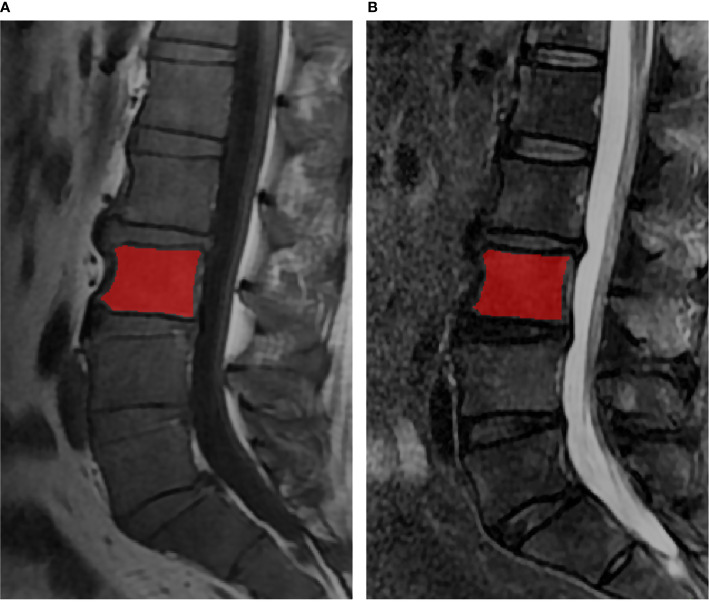
The lumbar MRI examination for multiple myeloma. The red regions were representative ROI segmentations of the vertebral bodies in T1WI **(A)** and T2WI **(B)**.

ITK-SNAP software v. 3.6.0 (www.itksnap.org) was used for manual segmentation (22). The regions of interest (ROIs) contained the whole bone marrow of vertebral bodies from L1 to L5, each slice was manually segmented by a musculoskeletal radiologist with 5 years of experience, while avoiding the cortical bone, the degeneration of the endplate and Schmorl’s nodes (**Figure 1**). All the ROIs were validated by another musculoskeletal radiologist with 13 years of experience.

### Radiomics Feature Extraction and Preprocessing

A total of 1316 radiomics features of each vertebral bodies were extracted from the T1WI and T2WI-FS based on the AK, including: 1) 18 first-order histogram features, 2) 14 shape features, 3) 75 texture features (24 gray-level co-occurrence matrix features, 14 gray-level dependence matrix features, 16 gray-level size-zone matrix features, 16 gray-level run-length matrix features, 5 neighboring gray-tone difference matrix features), 4) 744 wavelet features, by turning the ratio of weight to band-pass sub-bands (LLH, LHL, LHH, HLL, HLH, HHL) and low- and high-frequency sub-bands (LLL and HHH), and applied for each wavelet basis function, we obtained different information from images. 5) 279 local binary pattern features, with lbp3Dlevels of 2, lbp3DIcosphereRadius of 1, and lbp3DIcosphereSubdivision of 1, and 6) 186 Laplacian of Gaussian features, for which sigma value of 2 and 3 were used as filter parameters.

Radiomics features from the five vertebral bodies of the lumbar spine were summarized for each patient as mean values. Prior to the feature selection, all features were normalized by replacing the outliers with the median of the particular variance vector and standardizing the data using Z-score standardization method.

### Feature Selection and Radiomics Signature Construction for OS

121 patients were randomly divided into a training set (n = 84) and a validation set (n = 37) at a ratio of 7:3. Univariate cox regression analysis was first conducted to pick up those features with p value less than 0.05. Spearman correlation with a threshold of 0.8 was then applied to remove those features with high correlation. LASSO cox regression analysis with 5-fold cross-validation was finally used for multivariate feature selection. The LASSO regularization involved a parameter λ to control the number of selected features where a larger λ retains more features, and the final feature number was therefore determined by λ to maximize the C-index in the training set. The multiple-feature-based radiomics signature, that is, radiomics score (rad-score), was then calculated for each patient *via* a linear combination of selected features that were weighted by their respective coefficients.

The potential association of the radiomics signature with OS was first assessed in the training cohort and then validated in the validation cohort by using Kaplan-Meier survival analysis. The patients were classified into high or low risk groups in the training cohort, using the threshold of rad-score identified by the X-tile (23). Then, the same threshold value was applied to the validation cohort.

### Radiomics Nomogram Building and Assessment

Univariate and multivariate Cox proportional hazards analyses were performed for individual clinical features selection. For multivariate Cox proportional hazards model, the stepwise selection was used. Next, the independent clinical factors and radiomics signature were incorporated to create the radiomics nomogram. To quantify the discriminative performance of the nomogram, Harrell’s concordance-index (C-index) was measured. The value of C-index ranges from 0.5 to 1, and higher C-index indicated better predictive performance of the model. In addition, calibration curves were plotted to assess the goodness-of-fit of the radiomics nomogram and the performance of the nomogram was then validated in the validation cohort.

Our study also constructed four other models for OS prediction. One model was based on the radiomics signature alone, then the clinical model based on independent clinical risk factors, and the remaining two were based on D-S and the ISS respectively. The prognostic values of the radiomics nomogram and the other four models were compared.

### Potential Association of the OS-Based Rad-Score and the PFS

Our study evaluated the potential association between the OS-based rad-score and the PFS. The PFS of rad-score defined low and high risk group was compared by the Kaplan-Meier survival curves in the training and validation group. And the 1-, 2- and 3-year PFS rates was compared between the low and high risk groups.

### Statistical Analysis

Differences in distributions between the variables examined were assessed with the unpaired, 2-tailed χ2 test or the Fisher exact test as appropriate. The Kaplan-Meier survival curves and log-rank test were used to estimate the survival difference between the low and high risk groups. Univariate and multivariate analyses were performed using the Cox proportional hazards model. All statistical analyses were performed using R software (R Core Team, Vienna, Austria) v. 3.6.1. Packages of “glmnet” was implemented for LASSO cox regression, “Survival” was used for KM and calibration curve, Nomogram was plotted by “rms”, and the C-index values were compared across different models by “compareC”. A two-sided *P* value < 0.05 was considered significant.

## Results

### Patients

The characteristics of all included patients were listed in **Table 1**, and the detailed treatment combinations were shown in the supplementary materials. Of the 121 patients, the number of endpoint events was 66 (54.55%) in OS and 90 (74.38%) in PFS. The median OS and PFS were 52.13 months (CI, 39.97-82.43 months) and 26.47months (CI, 20.12-35.51 months), respectively.

**Table 1:** Baseline clinical characteristics of patients.

Characteristics	Entire cohort, N = 121, n (%)	Training, N = 84, n (%)	Validation, N = 37, n (%)	*P*
Age≥65 (years)	40 (33.06)	28 (33.33)	12 (32.43)	0.92
gender				
female	43 (35.54)	29 (34.52)	14 (37.84)	0.73
male	78 (64.46)	55 (65.48)	23 (62.16)	
Immunoglobulin type				
IgG	56 (46.28)	37 (44.05)	19 (51.35)	0.82
IgA	29 (23.97)	22 (26.19)	7 (18.92)	
IgD	6 (4.96)	4 (4.76)	2 (5.41)	
Light chain	30 (24.79)	21 (25.00)	9 (24.32)	
D-S staging				
II	21 (17.36)	16 (19.05)	5 (13.51)	0.46
III	100 (82.64)	68 (80.95)	32 (86.49)	
ISS staging				
I	21 (17.36)	11 (13.10)	10 (27.03)	0.09
II	39 (32.23)	31 (36.90)	8 (21.62)	
III	61 (50.41)	42 (50.00)	19 (51.36)	
Cytogenetic abnormalities				
1q21 gain	49 (40.50)	34 (40.48)	15 (40.54)	0.99
del (17p)	12 (9.92)	8 (9.52)	4 (10.81)	0.83
del (13q)	49 (40.50)	33 (39.29)	16 (43.24)	0.68
IgH translocations	67 (55.37)	40 (47.62)	27 (72.97)	0.01
BMPC≥60%	24 (19.83)	19 (22.62)	5 (13.51)	0.25
β2-MG≥5.5 mg/L	61 (50.41)	42 (50.00)	19 (51.35)	0.89
Hemoglobin ≤ 100 g/L	76 (62.81)	55 (65.48)	21 (56.76)	0.36
Platelet ≤ 150 g/L	52 (42.98)	39 (46.43)	13 (35.14)	0.25
LDH≥250u/L	21 (17.36)	15 (17.86)	6 (16.22)	0.83
Albumin≥35 g/L	62 (51.24)	42 (50.00)	20 (54.05)	0.68
CRE≥177 μmol/L	20 (16.53)	17 (20.24)	3 (8.11)	0.10
Calcium≥2.75 mmol/L	10 (8.26)	9 (10.71)	1 (2.70)	0.14
Treatment				
Proteasome inhibitor-based	84 (69.42)	56 (66.67)	28 (75.68)	0.31
IMiD-based	33 (27.27)	26 (30.95)	7 (18.92)	
IMiD+proteasome inhibitor	4 (3.31)	2 (2.38)	2 (5.41)	
New agents* applied	92 (76.03)	62 (73.81)	30 (81.08)	0.39
Undergone ASCT	29 (23.97)	20 (23.81)	9 (24.32)	0.95

Ig, Immunoglobulin; D-S, Durie-Salmon staging system; ISS, International Staging System; BMPC, bone marrow plasma cells; β2-MG, β2-microglobulin; LDH, lactate dehydrogenase; CRE, creatinine; IMiD, immunomodulating drugs; ASCT, autologous stem cell transplantation. *New agents, including bortezomib and lenalidomide.

### Construction of Radiomics Feature-Based Radiomics Signature

A total of sixteen significant radiomics features were extracted in the training set, with twelve from the T1WI and four from the T2WI. Of the sixteen features, four were local binary pattern features, seven Laplacian of Gaussian features, four wavelet features, and one shape features. The details were presented in **Table S1**.

**Figure 2:**
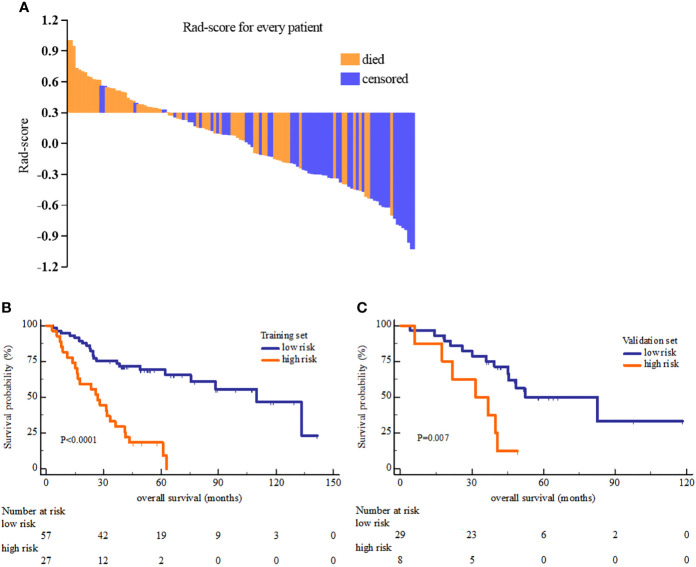
The Rad-score distribution of each patient **(A)** and the Kaplan-Meier overall survival analysis of high and low risk group in the training set **(B)** and validation set **(C)**. The *P* value of survival curves were generated by log-rank test.

Rad-score was constructed using the formula (supplementary materials). The rad-score distribution and survival status showed that patients usually had poorer survival with higher score than those with lower score (**Figure 1**). The optimal cutoff value of rad-score was 0.33 that generated by X-tile plot. Accordingly, patients were stratified into low risk group (rad-score<0.33) and high risk group (rad-score≥0.33). The survival analyses indicated a significant difference between the two groups both in the training (log-rank *P*<0.0001) and validation cohorts (log-rank *P*=0.007) (**Figure 2**).

### The Performance of Radiomics Nomogram and Comparison

Univariate cox proportional hazards analysis showed that there were eight clinical factors associated with OS, and multivariate cox analysis confirmed three independent clinical factors (**Table 2**). Furthermore, the radiomics signature was the mos important predictor of OS in multivariate analysis (HR=5.718, *P*<0.0001).

**Table 2:** Univariate and multivariate analysis of clinical risk factors associated with OS.

Variable	Univariate		Multivariate	
	HR (95% CI)	*P*	HR (95% CI)	*P*
BMPC≥60%	3.828 (2.194-6.678)	<0.001		
1q21 gain	3.054 (1.836-5.079)	<0.001	2.553 (1.506-4.330)	0.001
del (17p)	2.892 (1.453-5.755)	0.002	2.150 (1.069-4.327)	0.032
β2-MG≥5.5 mg/L	4.248 (2.446-7.376)	<0.001	2.789 (1.458-5.338)	<0.001
Hemoglobin ≤ 100 g/L	3.816 (2.075-7.016)	<0.001		
Platelet ≤ 150 g/L	1.990 (1.224-3.233)	0.005		
Albumin≥35 g/L	0.548 (0.335-0.897)	0.017		
CRE≥177 μmol/L	2.439 (1.355-4.388)	0.003		

HR, hazard ratio; CI, confidence interval; BMPC, bone marrow plasma cells; β2-MG, β2-microglobulin; CRE, creatinine.

**Figure 3:**
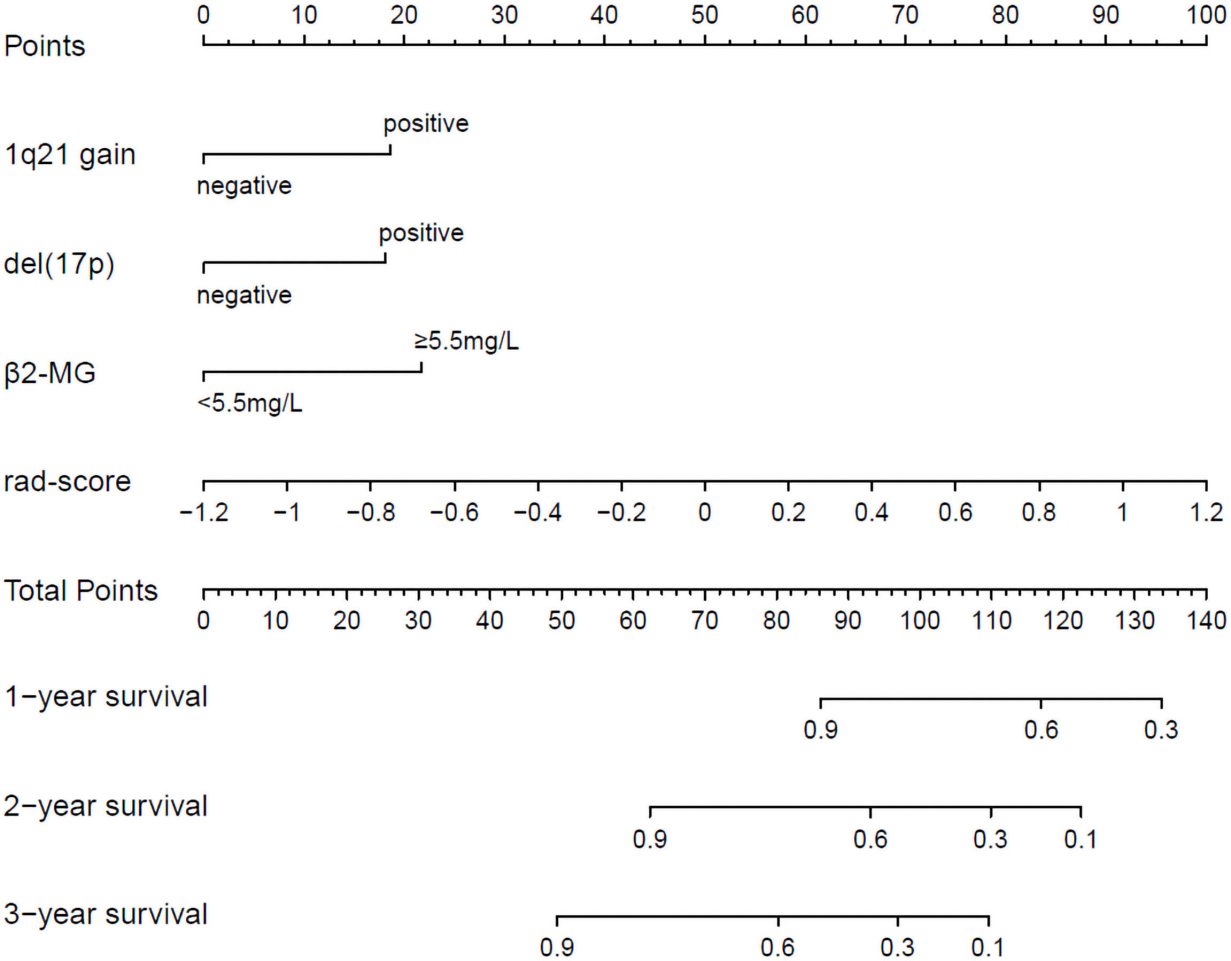
Radiomics nomogram for 1-, 2- and 3-year OS prediction. The 1q21 gain, del (17p), β2-MG≥5.5 mg/L and rad-score were the factors located on each axis. The patient receives a line drawn straight upward to the point axis of each factor. The points identified on the scale of each factor were summed to obtain a total point. For finding the patient’s probability of survival at 1-, 2- and 3-year, the line was drawn down.

**Figure 4:**
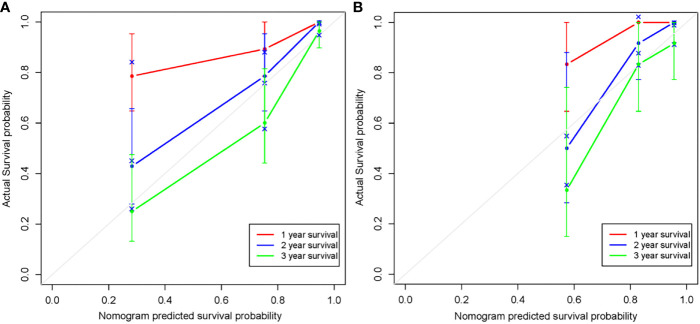
Calibration curves of the radiomics nomogram in the training set **(A)** and validation set **(B)**. The agreement between estimated and actual overall survival reflected the calibration power of the nomogram. Nomogram estimated survival time is plotted on the x-axis, and the actual survival time is plotted on the y-axis. The plot of 1-, 2- and 3-year survival close to the 45-degree line indicated good predictive ability of the nomogram.

The radiomics nomogram was generated by incorporating the three clinical factors and radiomics signature in the training set (**Figure 3**). Good discrimination performance of the nomogram was confirmed in the validation set (C-index:0.812, CI: 0.708,0.916). The calibration curves suggested a satisfactory agreement between the nomogram prediction and actual observation for 1-, 2- and 3-year OS, in both training and validation set (**Figure 4**). The other four models were constructed including the radiomics model based on radiomics signature alone, clinical model based on the three clinical predictors including β2-MG≥5.5 mg/L, 1q21 gain and del(17p), and the remaining two based on D-S and ISS staging system respectively. The performance of these five models was evaluated by the C-index in both the training and the validation cohorts (**Table 3**). In the training cohort, the radiomics nomogram (C-index, 0.793) was significantly better than the radiomics signature model (C-index, 0.742; P=0.014), clinics model (C-index, 0.733; P=0.022), ISS (C-index, 0.671; P<0.01) and D-S (C-index, 0.554; P<0.01). In the validation cohort, the radiomics nomogram (C-index, 0.812) was better than the other four models, but this trend reached statistical significance only when compared with the radiomics signature model (C-index, 0,717; P<0.01) and D-S (C-index, 0.512; P<0.01).

**Table 3:** Performance of radiomics nomogram and the other four models.

Model	Training cohort		Validation cohort	
	C-index	95% CI	C-index	95% CI
Radiomics nomogram	0.793	(0.730,0.856)	0.812	(0.708,0.916)
Radiomics signature	0.742	(0.651,0.834)	0.717	(0.576,0.858)
Clinics	0.733	(0.664,0.802)	0.799	(0.707,0.891)
ISS	0.671	(0.587,0.755)	0.761	(0.631,0.892)
D-S	0.554	(0.489,0.619)	0.512	(0.427,0.597)

C-index, index of concordance; CI, confidence interval; ISS, International Staging System; D-S, Durie-Salmon Staging System.

### Correlation Between OS-Based Radiomics Signature and PFS

**Figure 5:**
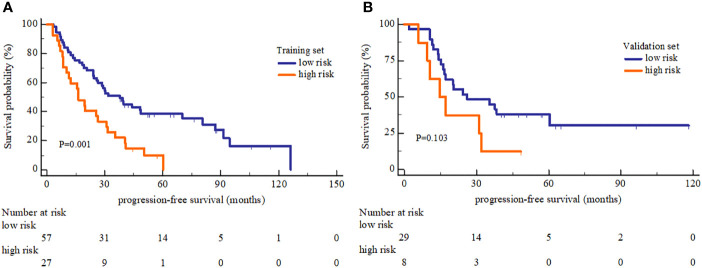
Kaplan-Meier Progression-free survival analysis of high and low risk group. **(A)** The PFS showed significant different between high and low risk group in the training set. **(B)** The PFS showed no significant different between high and low risk group in the validation set.

In PFS analysis, the high and low risk group defined by OS-based radiomics signature in the training set showed a significant split in the Kaplan-Meier survival curve (log-rank *P*=0.001), and a moderate split in the validation set (log-rank *P*=0.103) (**Figure 5**). The 1-, 2-, and 3-year PFS rate were all different between low and high risk group with statistically significant (**Table 4**).

**Table 4:** Different PFS rate in low and high risk group.

PFS rate	Low risk group n (%)	High risk group n (%)	χ2 value	*P*
1-year	70 (81.39)	22 (62.86)	4.691	0.03
2-year	54 (62.79)	14 (40.00)	5.249	0.02
3-year	42 (48.84)	7 (20.00)	8.585	0.003

## Discussion

In the present study, the radiomics signature, based on the extracted radiomics features from bone marrow MRI, had predictive ability in MM survival. The Kaplan-Meier survival analysis showed an obviously shorter OS in high risk group in comparison with low risk group, which was further confirmed in the validation group. The radiomics nomogram incorporating clinical factors and radiomics signature achieved a more accurate OS prediction than other models. In addition, the OS-based radiomics signature lacked the predictive power for PFS, but the OS-based radiomics signature had certain association with MM PFS. The bone marrow MRI radiomics was an important factor for predicting MM OS, the strong incremental effect on OS prediction may provide valuable information for ensuring proper clinical intervention measures.

The correlation of the MRI patterns and MM survival has been explored by many scholars, and a meta-analysis which summarized 10 studies elucidated a relationship may exist between MRI patterns and MM prognosis (24). The quantitative parameters of MRI were also considered valuable prognostic factors, and Maximilian et al. found the Kep-values, measured in dynamic contrast-enhanced MRI, were positively correlated to shorter OS in MM (15). Additionally, a recent prospective study indicated the baseline bone marrow ADC value of diffusion-weighted MRI can be seen as a potential independent predictor for MM survival (16). Nonetheless, the potentially useful information of MRI has yet not been fully exploited. Several studies have shown some efficiency of the bone marrow radiomics, a study had revealed that dual-energy CT textural features correlate well with MM-related serologic parameters and histology (25). Another study confirmed the predictive value of radiomics based on PET-CT imaging in MM (26). Kaspar et al. (27) focused on the alteration of textural features based on MRI before and after MM treatment, and confirmed the ability of textural features in assessing MM treatment response. A recent study demonstrated the satisfactory performance of radiomics to differentiate newly diagnosed myeloma lesions from metastatic lesions (28). Another radiomics analysis showed added value for MM pattern identification (29). Although the advantages of MRI in bone marrow infiltration and the potential ability of radiomics were obvious, few studies explored the role of bone marrow MRI radiomics in MM survival analysis.

In our study, a total of sixteen MRI radiomics features were selected for MM OS analysis and most of them from the T1WI. There was no doubt that T1WI plays an important role in MM analysis. As early as 2016, Zhou et al. (30) have reported that the dynamic intensity entropy transformation based only on T1WI could assess the treatment response of MM. T2WI with fat suppression that removed the interference from fatty hypointensity was widely used in MM diagnosis and prognosis (24, 27). However, there are few related researches on the prediction efficiency of MRI radiomics in MM. Though our result showed the limited value of T2WI-FS in MM survival prediction since the influence of T2WI-FS for radiomics signature building was relatively small, the application value of T2WI-FS in MM radiomic analysis should be further explored.

Aside from the radiomics signature, three clinical factors containing β2-MG≥5.5 mg/L, 1q21 gain and del(17p) also showed the prognostic value for MM survival in this study. The β2-MG was a classical risk factor for MM, that increased level of β2-MG reflects the high tumor burden and impairment of renal function, and the β2-MG with clear cut-off was confirmed as a powerful prognostic factor by the ISS system (6, 31). Cytogenetic abnormalities were prevalent in MM patients, correlating with a more proliferative myeloma and thus a particularly poor outcome (32, 33). Del(17p) was a strong poor prognostic factor, for it induces clonal immortalization and survival of tumor cells that negatively affect the MM survival (34). 1q21 gain is among the most common cytogenetic finding in MM, associated with relatively short PFS and OS even when treated with novel triplet regimens (35). The Mayo clinic risk stratification divided MM into a high risk group and standard risk group, and both the del (17p) and 1q21 gain were categorized as high risk factors (36). Others such as the elevated lactate dehydrogenase (LDH), decreased hemoglobin and platelet, the use of novel agent therapy and undergone ASCT, were common factors that influence the MM prognosis (36–38). However, these factors did not reach statistical significance in our study, and this may be due to the limited amount of data and the unavoidable selection bias.

The radiomics nomogram showed the highest C-index in this study, indicating the predictive ability of nomogram was not only better than the classic D-S and ISS, but also outperformed the clinical and radiomics signature models. In addition, it was obvious that both D-S and ISS showed relatively poor predictive ability, especially the D-S. This result was reasonable, for D-S was the first established staging system that mainly reflecting the tumor burden of MM, but the prognostic value was limited (5, 39). And the ISS, established using β2-MG and albumin, was widely used for risk stratification since 2005, but further improvement was needed (6, 40). We also found the performance of the radiomic signature for predicting OS was comparable to the clinical factors, but combining the radiomics signature and clinical factors improved the prediction accuracy. This suggested that the radiomics signature was valuable, it can provide independent and supplementary prognostic information. Moreover, radiomics signature may reflect some underlying pathophysiologic characteristics of MM, further study should explore the biological meanings at the molecular level. As the accurate prognostic prediction of MM patients is urgently needed in the era of new drugs, radiomics nomogram may has the potential for risk stratification. Patients with high risk should be treated with more advanced therapy for survival improvement.

In addition, we evaluated the prognostic power of the OS-based radiomics signature for PFS rather than constructing additional model for PFS prediction. The result showed the obvious difference of PFS between low and high risk group in the training set but no significant difference in the validation set, indicating that the predictive ability of OS-based radiomics signature for PFS was lacked. This was reasonable since the radiomics signature and its cut-off was originally obtained based on OS. For the1-, 2- and 3-year PFS rate, the differences between low and high risk group all achieved statistical significance. Indeed, there was a certain correlation between OS-based radiomics signature and PFS, which in part due to the fact that the PFS are linked to OS and often be used as a surrogate especially in clinical trials for the new drugs evaluation (41). The translatability of the signature indicated the prognosis-related endpoints may share some common radiomics features in MM, and the OS-based radiomics signature may also correlate with other prognosis-related endpoints such as treatment response and minimal residual disease status.

There were some limitations in this study besides for the inherent problems of retrospective design. First, this small single-center sample may not represent the general patient population, and the established nomogram should be validated by external multicenter data. Second, the ROI was manually delineated, which was laborious and time-consuming. The next step is to explore automatic segmentation for improving the clinical efficiency. Third, a substantial part of patients in our study lacked the baseline data of the IgH translocation t (11,14), t (4,14) and t (14,16), and these cytogenetic abnormalities might influence the MM survival in our study. Moreover, the Revised ISS cannot be analyzed due to these missing cytogenetic information, and further study is needed. Finally, only two routine MRI sequences were used for this radiomic analysis, and multi-parameter MRI may provide additional information that further improving the prognostic efficiency of the nomogram.

In conclusion, our study showed the developed radiomics nomogram may have the ability for MM OS prediction. Furthermore, the OS-based radiomics signature had certain association with MM PFS. These results indicated some prognostic efficiency of bone marrow MRI radiomics in MM, and this simple noninvasive method may have the potential for clinical risk stratification.

## Data Availability Statement

The original contributions presented in the study are included in the article/**Supplementary Material**. Further inquiries can be directed to the corresponding author.

## Ethics Statement

The studies involving human participants were reviewed and approved by the Institutional Ethics Committee in Peking University People’s Hospital. Written informed consent for participation was not required for this study in accordance with the national legislation and the institutional requirements.

## Author Contributions

Study management and guidance: NH. Study design: NH and YLi. Clinical data acquisition and analysis: YLi and YLiu. Imaging data acquisition and analysis: YLi, SW, and CS. Experimental studies: YLi, PY, and CH. Manuscript preparation: YLi, YLiu, and LC. Manuscript Review: NH.

## Funding

This work was supported by the National Natural Science Foundation of China (No.81971575).

## Conflict of Interest

Author SW was employed by GE Healthcare.

The remaining authors declare that the research was conducted in the absence of any commercial or financial relationships that could be construed as a potential conflict of interest.

## Publisher’s Note

All claims expressed in this article are solely those of the authors and do not necessarily represent those of their affiliated organizations, or those of the publisher, the editors and the reviewers. Any product that may be evaluated in this article, or claim that may be made by its manufacturer, is not guaranteed or endorsed by the publisher.
