# Supplementary material for: MRI-Based Bone Marrow Radiomics Nomogram for Prediction of Overall Survival in Patients With Multiple Myeloma
Source: Front Oncol. 2021 Dec 1;11:709813. doi: 10.3389/fonc.2021.709813 (PMC8671997; doi:10.3389/fonc.2021.709813)
Supplement: Supplementary file 1 [file DataSheet_1.docx]

Supplementary Material

# Supplementary formula

Rad-score calculation formula: Rad-score=0.08673×T1WI_ lbp_3D_k _firstorder_Median + 0.35527×T1WI_lbp_3D_k_glcm_Correlation + (-0.33544)×T1WI_lbp_3D_k_glszm_ZoneEntropy + 0.21971×T1WI_log_sigma_3D_firstorder_Skewness + (-0.10696)×T1WI_log_sigma_3D_glcm_InverseVariance + (-0.04194)×T1WI_log_sigma_3D_glrlm_LongRunLowGrayLevelEmphasis + (-0.01809)×T1WI_log_sigma_3D_firstorder_90percentile + 0.18491×T1WI_log_sigma_3D_glszm _SmallAreaLowGrayLevelEmphasis + (-0.26644)×T1WI_wavelet_HHH_glcm_Correlation + 0.16763×T1WI_wavelet_HLL_glszm_SmallAreaLowGrayLevelEmphasis + (-0.34409)×T1WI_wavelet_LLH_firstorder_Skewness + 0.25471×T2WI_ lbp_3D_k_gldm_DependenceNonUniformityNormalized + 0.07728×T2WI_ log_sigma_3D_firstorder_Kurtosis + (-0.21820)×T2WI_ log_sigma_3D_firstorder_Skewness + (-0.26917)×T2WI_original_shape_Flatness + 0.04962 ×T2WI_wavelet_LLH_glcm_Idmn.

# Supplementary Table and figure

## Supplementary Table

Table S1 Selected radiomics features correlated with OS and their HRs

| feature | sequences | HR |
| --- | --- | --- |
| lbp_3D_k _firstorder_Median | T1WI | 1.091 |
| lbp_3D_k_glcm_Correlation | T1WI | 1.427 |
| lbp_3D_k_glszm_ZoneEntropy | T1WI | 0.715 |
| log_sigma_3D_firstorder_Skewness | T1WI | 1.246 |
| log_sigma_3D_glcm_InverseVariance | T1WI | 0.899 |
| log_sigma_3D_glrlm_LongRunLowGrayLevelEmphasis | T1WI | 0.959 |
| log_sigma_3D_firstorder_90percentile | T1WI | 0.982 |
| log_sigma_3D_ glszm _SmallAreaLowGrayLevelEmphasis | T1WI | 1.203 |
| wavelet_HHH_ glcm _Correlation | T1WI | 0.766 |
| wavelet_HLL_ glszm _SmallAreaLowGrayLevelEmphasis | T1WI | 1.183 |
| wavelet_LLH_ firstorder_Skewness | T1WI | 0.709 |
| lbp_3D_k_gldm_DependenceNonUniformityNormalized | T2WI | 1.290 |
| log_sigma_3D_firstorder_Kurtosis | T2WI | 1.080 |
| log_sigma_3D_firstorder_Skewness | T2WI | 0.874 |
| original_ shape_Flatness | T2WI | 0.764 |
| wavelet_LLH_ glcm_Idmn | T2WI | 0.952 |

glcm, gray level co-occurrence matrix; glszm, gray level size zone matrix; glrlm, gray level run length matrix; gldm, gray level dependence matrix; HR, hazard ratio.

## Supplementary Figure

**Figure S1.** The patient selection process. MM, multiple myeloma; MRI, magnetic resonance imaging.

# The detailed treatment combinations

In our study, three regimens that recommended by the International Myeloma Working Group (IMWG) guidelines were used, including proteasome inhibitor-based regimen, immunomodulatory drug-based regimen, and immunomodulatory + proteasome inhibitor regimen.

Overall, eighty-four patients (69.42%) received a proteasome inhibitor-based regimen. The combinations were: bortezomib, cyclophosphamide, and dexamethasone; bortezomib, doxorubicin, and dexamethasone; bortezomib and dexamethasone. Thirty-three patients (27.27%) received an immunomodulatory drug-based regimen. The combinations were: lenalidomide and dexamethasone; thalidomide, cyclophosphamide, and dexamethasone; thalidomide and dexamethasone; thalidomide, doxorubicin, and dexamethasone; thalidomide, pirarubicin, and dexamethasone; four patients (3.31%) received an immunomodulatory + proteasome inhibitor regimen. The combinations were: bortezomib, lenalidomide, and dexamethasone; bortezomib, thalidomide, and dexamethasone.
